# The Roles of Type 2 Cytotoxic T Cells in Inflammation, Tissue Remodeling, and Prostaglandin (PG) D_2_ Production Are Attenuated by PGD_2_ Receptor 2 Antagonism

**DOI:** 10.4049/jimmunol.2001245

**Published:** 2021-06-01

**Authors:** Wentao Chen, Jian Luo, Yuan Ye, Ryan Hoyle, Wei Liu, Rowie Borst, Shamsah Kazani, Eric A. Shikatani, Veit J. Erpenbeck, Ian D. Pavord, Paul Klenerman, David A. Sandham, Luzheng Xue

**Affiliations:** * Respiratory Medicine Unit and National Institute for Health Research Oxford Biomedical Research Centre, University of Oxford, Oxford, United Kingdom;; † Novartis Institutes for BioMedical Research, Cambridge MA;; ‡ Novartis Pharma, AG, Basel, Switzerland; and; § Translational Gastroenterology Unit and Peter Medawar Building for Pathogen Research, University of Oxford, Oxford, United Kingdom

## Abstract

Human type 2 cytotoxic T (Tc2) cells are enriched in severe eosinophilic asthma and can contribute to airway eosinophilia. PGD_2_ and its receptor PGD_2_ receptor 2 (DP2) play important roles in Tc2 cell activation, including migration, cytokine production, and survival. In this study, we revealed novel, to our knowledge, functions of the PGD_2_/DP2 axis in Tc2 cells to induce tissue-remodeling effects and IgE-independent PGD_2_ autocrine production. PGD_2_ upregulated the expression of tissue-remodeling genes in Tc2 cells that enhanced the fibroblast proliferation and protein production required for tissue repair and myofibroblast differentiation. PGD_2_ stimulated Tc2 cells to produce PGD_2_ using the routine PGD_2_ synthesis pathway, which also contributed to TCR-dependent PGD_2_ production in Tc2 cells. Using fevipiprant, a specific DP2 antagonist, we demonstrated that competitive inhibition of DP2 not only completely blocked the cell migration, adhesion, proinflammatory cytokine production, and survival of Tc2 cells triggered by PGD_2_ but also attenuated the tissue-remodeling effects and autocrine/paracrine PGD_2_ production in Tc2 induced by PGD_2_ and other stimulators. These findings further confirmed the anti-inflammatory effect of fevipiprant and provided a better understanding of the role of Tc2 cells in the pathogenesis of asthma.

## Key Points

- Multiple proinflammatory effects of Tc2 cells are inhibited by DP2 antagonism.
- Tissue-remodeling functions of Tc2 cells are attenuated by DP2 antagonism.
- Autocrine/paracrine PGD_2_ production in Tc2 cells is reduced by DP2 antagonism.

## Introduction

Type 2 immunity plays critical roles in the pathogenesis of asthma, particularly in type 2–high asthma, as type 2 cytokines, such as IL-4, IL-5, and IL-13, are important drivers of many features of airway inflammation in the disease, including bronchoconstriction, airway eosinophilia, and IgE upregulation. Increased local concentrations of type 2 cytokines are detected in both airways and bronchoalveolar lavage fluid of patients with asthma (1). It is well accepted that type 2 cells, including Th2 and group 2 innate lymphoid cells (ILC2) are important players in type 2 immunity (2). We have reported recently that type 2 cytotoxic T cells (Tc2), another group of type 2 cells, are significantly enriched in the peripheral blood and airways of patients with severe eosinophilic asthma, which can contribute to eosinophilia directly or indirectly, suggesting a crucial role of this group of cells in the disease (3). However, the biology of Tc2 cells has not been studied as well as that of Th2 and ILC2 cells, and many potential roles of Tc2 cells in the pathogenesis of inflammatory diseases remain unclear.

All these type 2 cells highly express PGD_2_ receptor 2 (DP2), also known as chemoattractant receptor homologous molecule expressed on Th2 cells (CRTH2), a receptor for PGD_2_, which can be used as a marker of these type 2 cells, as depletion of DP2^+^ cells from CD4 or CD8 populations can almost completely remove all type 2 cytokine-producing cells in the populations (2–4). PGD_2_ is a major lipid mediator that is released from mast cells during an allergic response and is upregulated in asthma, according to disease severity (5–7). Two G protein–coupled receptors have been identified as PGD_2_ receptors, PGD_2_ receptor 1 (DP1), formerly known as DP, and DP2 (8, 9). However, DP2 is the dominant receptor mediating the effect of PGD_2_ in the above type 2 cells, as DP1 agonists or antagonists do not exhibit any obvious effect in these cells in vitro (10). Activation of DP2 elicits proinflammatory reactions in these cells, including cell migration, proinflammatory cytokine production, and suppression of apoptosis (3, 10–12). Therefore, inhibition of DP2 is considered as a potential approach to control type 2 immunity-mediated inflammatory diseases, and DP2 antagonists remain under clinical investigation (13, 14). Fevipiprant is a potent and selective DP2 antagonist that has shown therapeutic benefit in certain subsets of asthma patients in phase 2 clinical trials (15–17). In a mechanistic phase 2 clinical trial study in patients with persistent eosinophilic asthma, fevipiprant not only reduced airway inflammation but also improved epithelial integrity and reduced airway smooth muscle (ASM) mass (16, 17). In two recently published phase 3 studies, although neither trial showed a statistically significant reduction in asthma exacerbations, consistent and modest reductions in exacerbation rates were observed with a high dose of fevipiprant in both studies (18). However, the underlying anti-inflammatory mechanism of fevipiprant is still only known to a limited extent. An x-ray crystallographic study illustrated how fevipiprant competitively occupies a semioccluded ligand-binding pocket in DP2 to block the function of the receptor (19). Previous in vitro studies demonstrated the anti-inflammatory effect of fevipiprant in DP2-mediated reactions in Th2 and ILC2 cells (17, 20, 21). The effects of DP2 antagonism in Tc2 cells are not fully understood.

In this study, we explored some novel proinflammatory and profibrotic functions of Tc2 cells, focusing on protissue remodeling effects and PGD_2_ autocrine/paracrine production. Fevipiprant was used as a potent tool to dissect the role of the PGD_2_/DP2 axis and DP2 antagonism on these functions. Our observations provide further evidence of the important and pleiotropic roles of Tc2 cells in eosinophilic asthma and potential use of DP2 antagonism in type 2 inflammation.

## Materials and Methods

### Human clinical samples

Patients meeting the American Thoracic Society/European Respiratory Society definition of severe asthma with a sputum eosinophil count of >3% (eosinophilic) and healthy control subjects were recruited from John Radcliffe Hospital, Oxford, U.K. (22). The studies were approved by South Central–Oxford B Research Ethics Committee, Oxford, U.K. (18/SC/0361), and written informed consent was obtained from each donor before sample collection.

### Human CD8^+^DP2^+^ Tc2 cell preparation and treatment

Human Tc2 cells were isolated from fresh clinical blood samples collected in heparin-coated tubes or CD leukocyte cones (National Blood Service, Oxford, U.K.). PBMCs were prepared by gradient with Lymphoprep (STEMCELL Technologies), then CD3^+^CD8^+^CD4^−^DP2^+^ cells were sorted into 96-well plates using a BD FACSAria III sorter. Cells were amplified in culture for about a month with RPMI 1640 supplemented with 10% human serum, 250 IU/ml IL-2, 50ng/ml anti-CD3 Ab, and irradiated feeder PBMCs. The purity (CD3^+^CD8^+^CD4^−^DP2^+^ cells >90%) of the cells after expansion and their response to PGD_2_ was confirmed with flow cytometry and chemotaxis before use.

For cytokine or other protein production assays, Tc2 cells were incubated with 100–200 nM PGD_2_ in presence or absence of various concentrations of fevipiprant (Novartis Pharma), BW245C, BW868C, or TM30089 (Cayman Chemical), as indicated in the *Results*, for 4 h at 37°C. The cell supernatants were harvested for ELISA or Luminex assays, and the cell pellets were stored at −80°C for quantitative RT-PCR (qRT-PCR) and microarray.

For PGD_2_ and leukotriene E4 (LTE_4_) assays, Tc2 cells were treated with 400 nM 13,14-dihydro-15-keto-PGD_2_ (DK-PGD_2_) or 5 µg/ml anti–CD3/CD28 Abs for varying lengths of time in the absence or presence of 1 µM fevipiprant or after 1-h preincubation with 10 µM diclofenac (Sigma-Aldrich), 10 µM flurbiprofen (Abcam), and 1 µM hematopoietic PGD synthase (hPGDS) inhibitor I (Cayman Chemical) or 20-min preincubation with 30 µM arachidonyl trifluoromethyl ketone (ATK; Cayman Chemical) or 10 µM U-73122 (Cayman Chemical). The cell supernatants were harvested for enzyme immunoassay assays, and the cell pellets were stored at −80°C for qRT-PCR.

### Fibroblast and Tc2 coculture

Human fibroblast cell line MRC-5 cells (American Type Culture Collection) were seeded in a 96-well plate at 1 × 10^3^ cells per well in MEM (Life Technologies) supplemented with 10% FBS and cultured overnight. After a gentle wash with PBS, 1 × 10^4^ Tc2 cells were loaded on the top of fibroblasts in a final volume of 200 μl of MEM containing 2% FBS in the presence or absence of 200 nM PGD_2_ or/and 1 µM fevipiprant. As a negative control, 200 µl of the same media were added into fibroblast cultures without Tc2 cells. In some experiments, Tc2 cells were loaded into a 0.5-µm filter insert on the top of the wells containing MRC-5. Otherwise, Tc2 conditioned media, after treatment with 200 nM PGD_2_ in the presence or absence of 1µM fevipiprant, or MEM, containing various concentrations of IL-4/13, were loaded instead of Tc2 cells. Images of the cell cultures were captured at different time points by using the IncuCyte ZOOM (Essen Bioscience). Confluency of fibroblasts was quantified as area of fibroblasts from images using the Trainable Weka Segmentation, a plugin in Fiji for ImageJ (21). The supernatants of the MRC-5 cultures were harvested for ELISA, and the cell pellets were collected for qRT-PCR analysis.

### Chemotaxis

Tc2 cells were resuspended in RPMI 1640 containing 10% human serum and then incubated with various concentrations of fevipiprant, BW245C, BW868C, TM830089, or medium for 1 h at 37°C in the upper chamber (5-µm pore size) of HTS Transwell-96 permeable supports (Corning). The upper chamber was then placed into the lower chamber of the supports containing prewarmed media supplemented with 100 nM PGD_2_, 1 µM BW245C, or medium for a further 1-h incubation. The cell migration to the lower chamber was quantified using an IncuCyte ZOOM.

### ELISA

The levels of IL-4, IL-5, IL-13, TGF-β1 (Invitrogen) or collagen I α1 (Bio-Techne) in the supernatants after the cell treatments were assayed with ELISA kits, according to the manufacturer’s instructions. The concentrations of PGD_2_ and LTE_4_ in the supernatants were measured with a PGD_2_–MOX enzyme immunoassay kit and LTE_4_ enzyme immunoassay kit (Cayman Chemical), respectively, according to the manufacturer’s instructions. The results were measured in an EnVision Multilabel Plate Reader (PerkinElmer).

### Luminex

The level of IL-3/8, CCL4, FasL, Galectin-3, GM-CSF, M-CSF, and TNF-α in the supernatants after the cell treatments were measured with Human Magnetic Luminex Performance Assay Base Kits (Bio-Techne) following the manufacturer’s instructions. The results were obtained with a Bio-Plex 200 system (Bio-Rad Laboratories).

### qRT-PCR

Total RNA from the Tc2 cell pellets or MRC-5 cells was extracted using RNeasy Mini Kit (QIAGEN). cDNA was prepared using a High-Capacity cDNA Reverse Transcription Kit (Applied Biosystems). qRT-PCR was conducted using Master Mix and Probes (Roche) or Fast SYBR Green Master Mix (Applied Biosystems) in a CFX96 Real-Time PCR Detection System (Bio-Rad Laboratories) (Supplemental Table I). *GAPDH* was used as a reference gene.

**Table I.** Study subjects (mean ± SD)

	Severe Eosinophilic Asthma (*n* = 6)	Healthy Control (*n* = 4)
Age (y)	54 ± 19	36 ± 12
Sex (male/female)	4/2	1/3
Blood eosinophils (cells/µl)	528.33 ± 272.28	NA
FEV1 (L/min)	2.39 ± 0.82	NA
FeNO (ppb)	37.17 ± 21.44	NA
BMI (kg/cm^2^)	29.90 ± 8.29	NA
Prednisolone use (yes/no)	1/5	0/4

BMI, body mass index; FeNO, fractional exhaled NO; FEV1, forced expiratory volume in 1 s; NA, not applicable; ppb, parts per billion.

### Microarray

RNAs from cultured Tc2 cells treated with or without PGD_2_ for 4 h were extracted with an RNeasy Mini Kit, and microarrays were conducted using an Illumina HumanHT-12 v4.0 Gene Expression BeadChip at the Transcriptomics Core Facility, The Jenner Institute, University of Oxford, Oxford, U.K. Preprocessing data analysis was performed using R Bioconductor packages. Genes involved in tissue-remodeling functions in Gene Ontology Terms 0060429, 0030198, 0048771, and 0042060 that reached an absolute log_2_ fold change >1 and were significant at *p* < 0.05 were selected using the Limma package (23). The heatmap was generated using pheatmap (https://CRAN.R-project.org/package=pheatmap) package.

### Apoptosis

Tc2 cells were incubated in RPMI 1640 in the presence or absence of 1 µM PGD_2_ or/and 1 µM fevipiprant for 12 h at 37°C. Cells treated with normal culture medium containing human serum were used as control. The cells were harvested and stained with annexin V (BioLegend) in an Annexin V Binding Buffer (BioLegend) following the BioLegend instructions. Results were acquired with a LSR II flow cytometer (BD Biosciences).

### Cell aggregation analysis

Cell aggregation was photographed using a Nikon Eclipse TS100 microscope. Images were analyzed with the Trainable Weka Segmentation. Briefly,cell clumps were identified using the Otsu method of thresholding. The intensity and size of identified objects were measured. Results are reported as integrated intensity.

### Flow cytometry

For the ex vivo cytokine production assays, PBMCs isolated from fresh blood of the patients with severe eosinophilic asthma were incubated with 1 µM PGD_2_, 1 µM fevipiprant, or in combination for 6 h at 37°C. Brefeldin A (5 µg/ml, BioLegend) was added 30 min after starting stimulation. The cells were then stained with an Ab panel against CD3, CD4, and CD8 with Zombie Aqua dye (BioLegend) followed by fixation with IC Fixation Buffer (Invitrogen) overnight at 4°C. The cells were treated with permeabilization buffer (Invitrogen) followed by intracellular staining with anti-human IL-13 and IL-5 (BioLegend) Abs. CD3^+^CD4^-^CD8^+^IL-5/13^+^ cells were detected with flow cytometer.

For hPGDS staining, fresh blood was labeled with the Ab panel against CD3, CD4, and CD8 with Zombie Aqua dye followed by RBC lysis with a FACS lysing solution (BD Biosciences) and cell permeabilization with permeabilization buffer and then intracellularly stained with Ab to hPGDS (a kind gift from Advanced Technology and Development, BML, Saitama, Japan).

For cyclooxygenase (COX) staining in cultured Tc2 cells, the cells were treated with 400 nM DK-PGD_2_ or 5 µg/ml anti–CD3/CD28 Abs in the absence or presence of 1 µM fevipiprant for 4 h, then fixed and permeabilized using a FOXP3 Fix/Perm Buffer Set (BioLegend) followed by intracellular staining with anti-human COX-1 and COX-2 Abs (BD Biosciences).

For DP2 endocytosis analysis, cultured Tc2 cells with or without preincubation with 10 µM flurbiprofen or 1 µM hPGDS inhibitor 1 for 1 h were stimulated with 200 nM PGD_2_ or 5 µg/ml anti–CD3/CD28 Abs in the absence or presence of 1 µM fevipiprant for 4 h and then stained with anti-DP2 Ab together with Zombie Aqua.

Results of the above staining were acquired with BD LSRFortessa or LSR II flow cytometers.

### Statistics

Data were analyzed by using one-way ANOVA, followed by a Tukey test. All *p* values <0.05 were considered statistically significant.

## Results

### DP2-mediated Tc2 cell migration, aggregation, and survival were inhibited by fevipiprant

To study the proinflammatory roles of human Tc2 cells in vitro, CD3^+^CD8^+^DP2^+^ Tc2 cells were isolated from the blood of healthy or severe eosinophilic asthma donors and expanded. Because Tc2 cells are enriched in the airways of eosinophilic asthma patients (3), we first examined the cell reactions that potentially contribute to cell recruitment and enrichment, including cell migration, adhesion, and survival.

**FIGURE 1.**
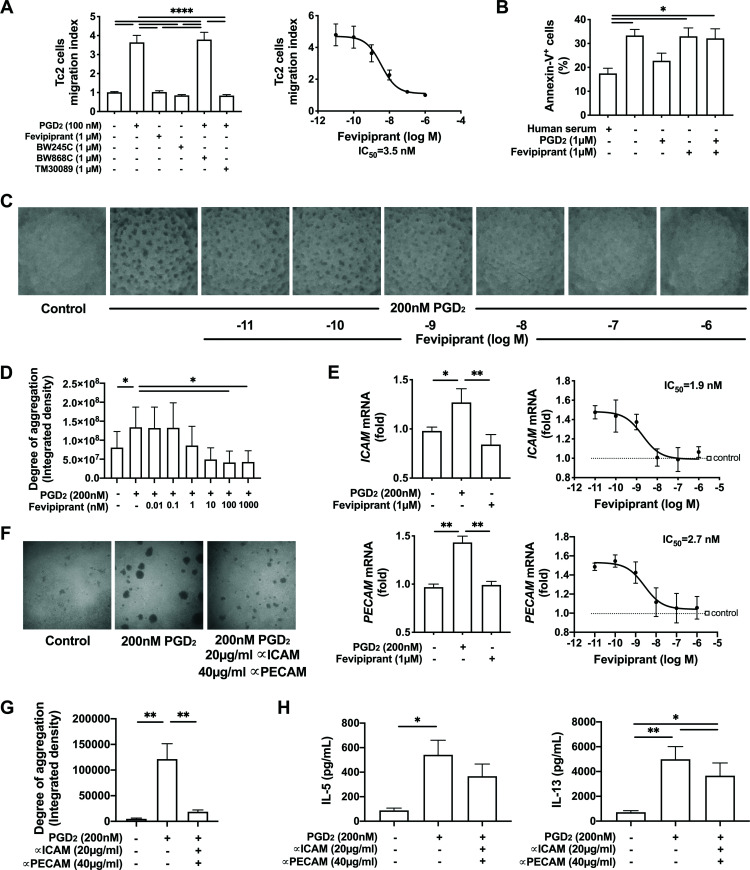
PGD_2_-induced cell migration, survival, and aggregation in cultured Tc2 cells from leukocyte cones were inhibited by fevipiprant. (**A**) Tc2 cell migration to 100 nM PGD_2_ in the presence or absence of 1 µM BW245C (DP1 agonist), 1 µM BW868C (DP1 antagonist), 1 µM TM30089 (DP2 antagonist), or various concentrations of fevipiprant as determined by chemotaxis assay. (**B**) Annexin-V^+^ apoptotic Tc2 cells induced by human serum deprivation in the presence or absence of 1 µM PGD_2_ and/or 1 µM fevipiprant as detected with flow cytometry. (**C** and **D**) Tc2 cell aggregation after incubation with 200 nM PGD_2_ in the absence or presence of various concentrations fevipiprant for 2 h, as recorded under microscopy (C) and then quantified with ImageJ (D). (**E**) mRNA levels of *ICAM* and *PECAM* after PGD_2_ stimulation in the absence or presence of various concentrations of fevipiprant as examined with qRT-PCR. (**F** and **G**) Cell aggregation after incubation with 200 nM PGD_2_ in the absence or presence of neutralization Abs to ICAM (20 µg/ml) and PECAM (40 µg/ml) as recorded under microscopy (F) and then quantified with ImageJ (G). (**H**) Type 2 cytokine production after PGD_2_ stimulation with or without neutralization Abs to ICAM/PECAM determined by ELISA. (C) Original mgnification ×240; (F) original magnification ×200. Data are expressed as mean ± SEM of 10 (A), 5 (B), 7 (D), 3 (E), 4 (G), or 6 (H) independent experiments. *p < 0.05, **p < 0.01, ****p < 0.0001.

PGD_2_ (100 nM) but not DP1 agonist BW245C induced Tc2 cell migration strongly in a chemotaxis assay, which was inhibited by DP2 antagonist TM30089 but not DP1 antagonist BW868C, suggesting that the migration was mediated specifically by DP2 (Fig. 1A). Fevipiprant inhibited PGD_2_-induced (100 nM) Tc2 migration in a dose-dependent manner with an IC_50_ = 3.5 ± 3.6 nM.

We next examined the effect of DP2 on Tc2 cell survival. Cell apoptosis was increased after serum deprivation in the cell culture (Fig. 1B). PGD_2_ (1 µM) suppressed apoptosis, which was reversed by fevipiprant (1 µM, (Fig. 1B).

Cell aggregation is a marker of cell adhesion in in vitro culture. Aggregation of Tc2 cells rapidly occurred within 1 h after PGD_2_ (200 nM) treatment, which persisted for 2–4 h (Fig. 1C), and was inhibited by fevipiprant in a dose-dependent manner (Fig. 1C, 1D). This aggregation was dominantly mediated by the adhesion molecules ICAM and PECAM, as the expression of these molecules was upregulated by PGD_2_ stimulation and inhibited by fevipiprant (Fig. 1E), and neutralization Abs to ICAM and PECAM significantly reduced the cell aggregation (Fig. 1F, 1G). Interestingly, inhibition of Tc2 aggregation with these neutralization Abs also reduced IL-5 and IL-13 production from the cells (Fig. 1H), suggesting that type 2 cytokine production in Tc2 is partly dependent on ICAM and PECAM expression.

### DP2-mediated proinflammatory cytokine production in Tc2 cells was inhibited by fevipiprant

**FIGURE 2.**
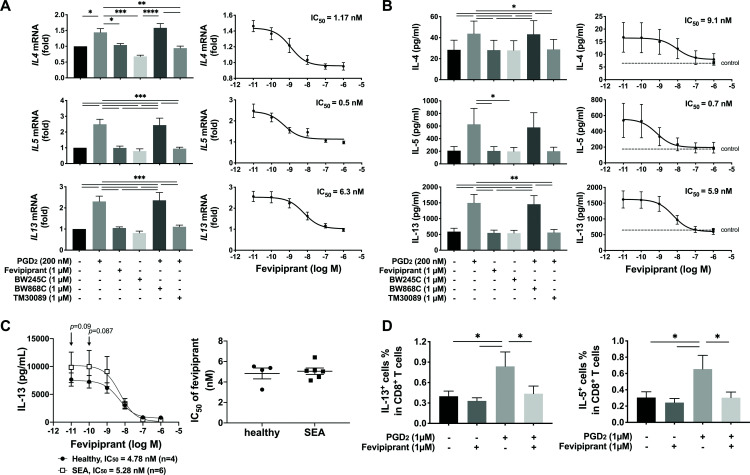
DP2-mediated type 2 cytokine production in Tc2 cells was blocked by fevipiprant in vitro and ex vivo. (**A**) mRNA or (**B**) protein levels for type 2 cytokines in cultured Tc2 cells from leukocyte cones after treatments with 200 nM PGD_2_ in the presence or absence of BW245C, BW868C, TM30089, or various concentrations of fevipiprant as measured with qRT-PCR or ELISA, respectively. (**C**) Effect of fevipiprant on IL-13 production induced by PGD_2_ (200 nM) in cultured Tc2 cells derived from healthy controls or severe eosinophilic asthmatics (SEA). IC_50_ values of fevipiprant were compared between the donor groups. (**D**) IL-5– and IL-13–producing Tc2 cells induced by PGD_2_ and/or fevipiprant in fresh blood from severe asthmatics as detected by using intracellular staining for flow cytometry. Data are expressed as mean ± SEM of 5 (A and C), 9–19 (B), or 4–6 (D) independent experiments. *p < 0.05, **p < 0.01, ***p < 0.001, ****p < 0.0001.

Type 2 cytokine (IL-4, IL-5, and IL-13) production was significantly upregulated in Tc2 cells in response to PGD_2_ mediated specifically by DP2, and was inhibited by TM30089 but not BW868C (Fig. 2A, 2B). Fevipiprant inhibited the response in a dose-dependent manner for both transcriptional mRNA (IC_50_ = 1.17 nM for *IL4*, 0.5 nM for *IL5*, and 6.3 nM for *IL13*) and translational protein (IC_50_ = 9.1 nM for IL-4, 0.7 nM for IL-5, and 5.9 nM for IL-13) levels.

We also compared type 2 cytokine production from cultured Tc2 cells between healthy donors and patients with severe eosinophilic asthma (Table I). The cells derived from asthmatics released slightly more IL-13 in response to PGD_2_ than those from healthy donors (Fig. 2C, left panel). The maximum responses achieved by Tc2 from healthy blood were only 72% of that achieved by Tc2 from asthmatic donors. However, no difference of the potency of fevipiprant was detected between healthy and asthmatic Tc2 (Fig. 2C, right panel).

To confirm the effects of PGD_2_ and fevipiprant on type 2 cytokine production in Tc2 cells under physiological conditions, we tested the responses in fresh blood from patients with severe asthma using intracellular staining for type 2 cytokines ex vivo (Fig. 2D). CD3^+^CD4^−^CD8^+^IL-5^+^/IL-13^+^ T cells were increased in the blood after PGD_2_ (1 µM) treatment. Coincubation with fevipiprant (1 µM) significantly blocked the increase. In contrast, it was difficult to detect type 2 cytokine–positive CD8^+^ T cells in the blood from healthy donors in response to PGD_2_.

**FIGURE 3.**
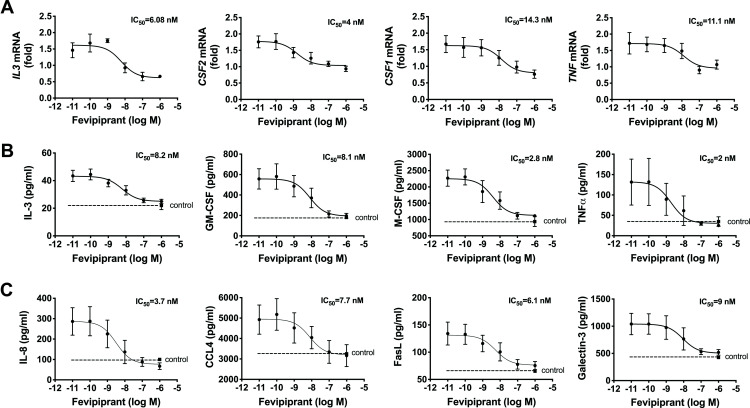
PGD_2_-induced proinflammatory cytokine production in cultured Tc2 cells from leukocyte cones was inhibited by fevipiprant. (**A**) mRNA or (**B**) protein levels for IL-3, GM-CSF, M-CSF, and TNF-α, and (**C**) protein levels for IL-8, CCL4, FasL, and Galectin-3 from Tc2 cells after treatments with 200 nM PGD_2_ in the presence of various concentrations of fevipiprant and measured with qRT-PCR (A) or Luminex (B and C), respectively. Data are expressed as mean ± SEM of six independent experiments.

PGD_2_ also upregulated production of many other proinflammatory cytokines, including IL-3, GM-CSF, M-CSF, and TNF-α at both mRNA and protein levels in Tc2 cells (Fig. 3A, 3B). The production of these cytokines was ablated by fevipiprant in a dose-dependent manner, with an IC_50_ = 6.08 nM (mRNA) and 8.2 nM (protein) for IL-3, an IC_50_ = 4 nM (mRNA) and 8.1 nM (protein) for GM-CSF, an IC_50_ = 14.3 nM (mRNA) and 2.8 nM (protein) for M-CSF, and an IC_50_ = 11.1 nM (mRNA) and 2 nM (protein) for TNF-α. Such an inhibitory effect of fevipiprant was also observed in some other proteins, such as IL-8, CCL4, FasL, and Galectin-3 (Fig. 3C).

### Tissue-remodeling effect of Tc2 cells induced by PGD_2_ was abolished by fevipiprant

**FIGURE 4.**
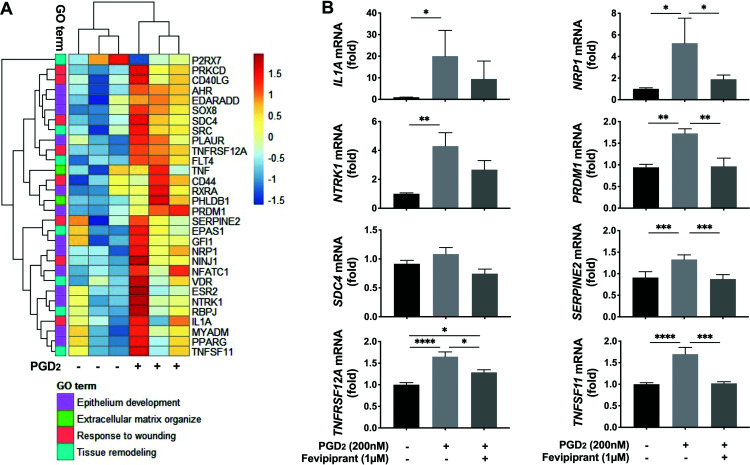
Upregulation of tissue-remodeling genes in cultured Tc2 cells from leukocyte cones by PGD_2_ was inhibited by fevipiprant. (**A**) Heat map showing tissue-remodeling genes significantly, defined as *p* < 0.05, upregulated by PGD_2_ (100 nM) in cultured Tc2 cells detected by microarray. (**B**) The upregulation of tissue-remodeling genes by PGD_2_ was inhibited by fevipiprant. The mRNA levels of tissue-remodeling genes in cells after treatment with PGD_2_ (200 nM) in the presence or absence of fevipiprant (1 µM) and measured with qRT-PCR. Data are expressed as mean ± SEM of three (A) or six to seven (B) independent experiments. *p < 0.05; **p < 0.01, ***p < 0.001, ****p < 0.0001.

Airway remodeling is a pathophysiological feature of asthma. To further understand the pathogenic role of Tc2 and PGD_2_ on airway remodeling, we investigated the transcripts associated with tissue remodeling in response to PGD_2_ (100 nM) using microarrays (Fig. 4A). The data showed that a large number of tissue-remodeling genes expressed in Tc2 cells were upregulated by PGD_2_. Prominent among these were aryl hydrocarbon receptor (*AHR*), ectodysplasin-A receptor-associated adapter protein (*EDARADD*), endothelial PAS domain-containing protein 1 (*EPAS1*), fms-related tyrosine kinase 4 (*FLT4*), heme oxygenase 1 (*HMOX1*), IL-1α (*IL1A*), neuropilin-1 (NRP1; *NRP1*), tropomyosin receptor kinase A (TrkA; *NTRK1*), urokinase receptor [uPAR] (*PLAUR*), peroxisome proliferator-activated receptor gamma (*PPARG*), PR domain zinc finger protein 1 (*PRDM1*), syndecan-4 (*SDC4*), glia-derived nexin (*SERPINE2*), TNF-α (*TNF*), receptor activator of NF-κΒ ligand (*TNFSF11*) and TWEAK receptor or fibroblast growth factor-inducible 14 (Fn14; *TNFRSF12A*). These genes could potentially promote epithelium development, fibroblast proliferation, extracellular matrix organization, and tissue remodeling. The results from the microarray were confirmed with qRT-PCR (Fig. 3, 4B; Supplemental Fig. 1A), with fevipiprant (1 µM) treatment at least partially inhibiting the gene upregulation.

**FIGURE 5.**
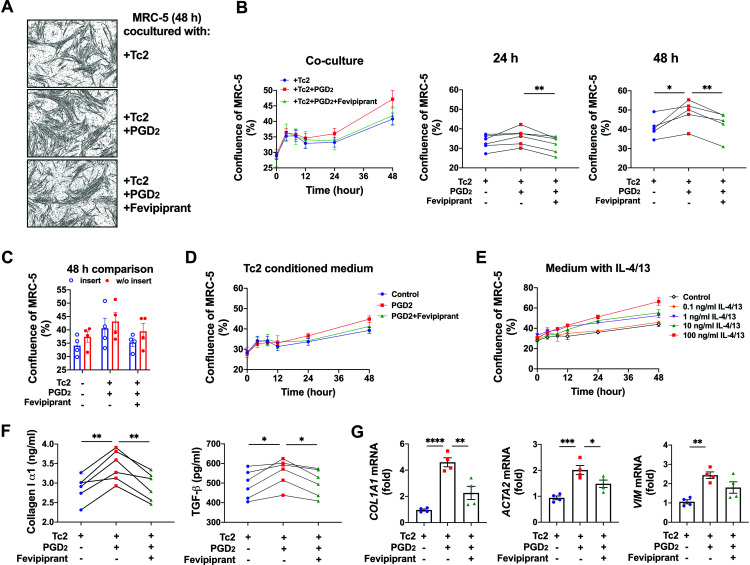
The tissue-remodeling effect of Tc2 cells (cultured from leukocyte cones) induced by PGD_2_ was abolished by fevipiprant. (**A** and **B**) Promoting effect of Tc2 cells on MRC-5 cell growth induced by PGD_2_ was abolished by fevipiprant. The confluence of MRC-5 cells in the coculture with Tc2 cells in a control medium, a medium containing 200 nM PGD_2_,or a medium containing 200 nM PGD_2_ and 1 µM fevipiprant for varying time lengths was recorded with IncuCyte (A) and quantified with Fiji (B). (**C**) Comparison of MRC-5 cell confluence in the cocultures using and without using inserts to separate the two types of cells at 48-h time point. (**D** and **E**) The confluence of MRC-5 cells after being cultured with the supernatants from Tc2 cells stimulated with PGD_2_ in the presence or absence of fevipiprant (D) or with MEM containing different concentrations of IL-4 and IL-13 (E). (**F**) Concentration of collagen I α1 and TGF-β in the supernatants of MRC-5 cells cocultured with Tc2 cells detected with ELISA. (**G**) mRNA levels of *COL1A1*, *ACTA2*, and *VIM* in MRC-5 cells cocultured with Tc2 cells as measured with qRT-PCR. (A) Original magnification ×100. Data are expressed as mean ± SEM of five to six (B), four (D), or three (E) independent experiments. *p < 0.05; **p < 0.01, ***p < 0.001, ****p < 0.0001.

To further confirm the potential physiological effect of the above genes in tissue remodeling, we conducted a fibroblast–Tc2 coculture assay (Fig. 5A, 5B). MRC-5 cells, a fibroblast cell line, were cultured with Tc2 cells in the presence or absence of PGD_2_ (200 nM) with or without fevipiprant (1 µM). The images of the cell cultures were recorded at different time points (Fig. 5A), then the confluence of MRC-5 cells was calculated (Fig. 5B). After a 24-h culture, Tc2 cells with PGD_2_ significantly enhanced the MRC-5 confluence compared with the culture without PGD_2_. Addition of fevipiprant together with PGD_2_ in the MRC-5-Tc2 coculture removed the enhancement, indicating the key role of PGD_2_/DP2 axis in this reaction. This enhancement was not due to the direct interaction between PGD_2_ and MRC-5 cells, as no such enhancement was observed in the same cultures without Tc2 cells (Supplemental Fig. 1B). Both soluble tissue-remodeling factors in the Tc2 medium and direct cell contact of MRC-5–Tc2 seem to contribute to the enhancement, as either using a filter insert to separate two types of cells in the coculture (Fig. 5C, Supplemental Fig. 1C) or culturing MRC-5 in a conditioned medium from activated Tc2 cells (Fig. 5D) still enhances MRC-5 confluence, but the level of the enhancement was slightly reduced compared with the coculture without insert (Fig. 5C, Supplemental Fig. 1C). Furthermore, in addition to the aforementioned tissue-remodeling proteins, type 2 cytokines could also enhance MRC-5 proliferation, as addition of IL-4 and IL-13 in MEM increased MRC-5 confluence in a dose-dependent manner (Fig. 5E), although the concentration of IL-13 (0.01–0.1 ng/ml) in the supernatants of cocultures or Tc2 conditioned medium (IL-4 was undetectable) was not enough to trigger the full enhancement (Supplemental Fig. 1D).

Increased levels of collagen I α1 and TGF-β1 were detected in the supernatant of MRC-5 cocultures with Tc2 in the presence of PGD_2_ (Fig. 5F), and upregulations of gene transcription for collagen I α1 (*COL1A1*), actin α2 (*ACTA2*), and vimentin (*VIM*) were also detected in the MRC-5 cells cocultured with Tc2 and PGD_2_ (Fig. 5G) that were inhibited by fevipiprant. These upregulations were not due to the direct interaction between PGD_2_ and MRC-5 cells, as such reactions were not detected in the same cultures without Tc2 cells (Supplemental Fig. 1E, 1F). These genes were not regulated by PGD_2_ in Tc2 cells, based on the microarray results.

### Production of PGD_2_ in Tc2 cells was attenuated by fevipiprant

**FIGURE 6.**
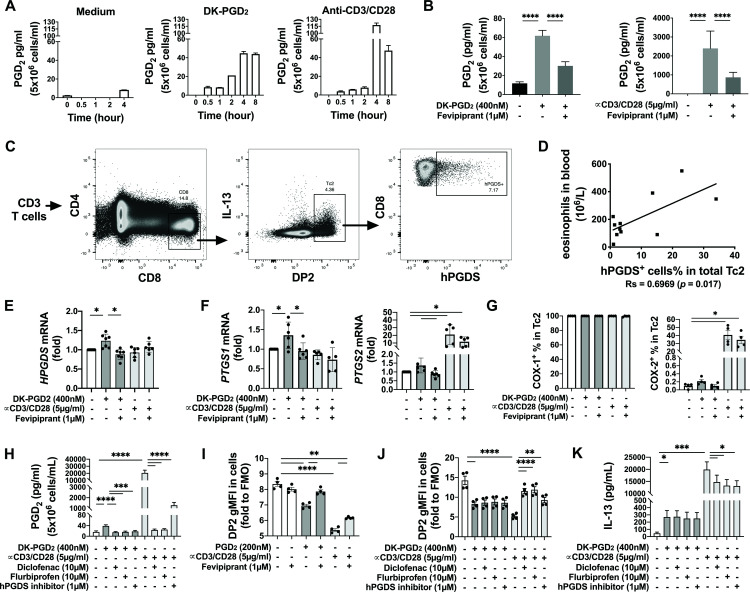
Autocrine PGD_2_ production in cultured Tc2 cells from leukocyte cones was attenuated by inhibition of DP2, hPGDS, and COXs. (**A**) PGD_2_ production from cultured Tc2 cells after stimulation with DK-PGD_2_ (400 nM) or anti–CD3/CD28 (5µg/ml) at different time points measured with MOX enzyme immunoassay. (**B**) Levels of PGD_2_ production after stimulation with DK-PGD_2_ or anti–CD3/CD28 in the presence or absence of fevipiprant for 4 h. (**C**) Gating strategy to detect hPGDS-positive Tc2 cells in fresh blood with flow cytometry. (**D**) Correlation of the level of hPGDS^+^ Tc2 cells and blood eosinophil counts in asthmatic patients. (**E** and **F**) The mRNA levels of *HPGDS* (E), *PTGS1* or *PTGS2* (F) in cultured Tc2 cells after treatments with DK-PGD_2_ or anti–CD3/CD28 Abs in the absence or presence of fevipiprant for 4 h as determined with qRT-PCR. (**G**) Expression of COX-1 and COX-2 in cultured Tc2 cells after treatments with DK-PGD_2_ or anti–CD3/CD28 Abs in the absence or presence of fevipiprant and measured with flow cytometry. (**H**) PGD_2_ production in cultured Tc2 cells after preincubation with diclofenac, flurbiprofen, or hPGDS inhibitor I for 1 h, followed by stimulation with DK-PGD_2_ or anti–CD3/CD28 for 4 h. (**I** and **J**) Comparison of DP2 levels on Tc2 cell surface after stimulation with PGD_2_ or anti–CD3/CD28 for 4 h in the absence or presence of (I) fevipiprant, (J) diclofenac, flurbiprofen, or hPGDS inhibitor I as determined with flow cytometry. (**K**) IL-13 production in Tc2 cells treated with PGD_2_ or anti–CD3/CD28 in the absence or presence of diclofenac, flurbiprofen, or hPGDS inhibitor I. Rs indicate Pearson correlation coefficients (two-tailed). Data are expressed as mean ± SEM of 3 (A), 6 (B), 10 (H), or 4 (K) independent experiments. *p < 0.05; **p < 0.01; ***p < 0.001; ****p = 0.0001.

The level of PGD_2_ is increased in the airways of asthmatics (3, 7). To investigate whether Tc2 cells are capable of contributing to PGD_2_ upregulation in the disease, we examined PGD_2_ production in Tc2 cells activated through DP2 or TCR. To enable differentiation of stimulator and product PGD_2_ in the PGD_2_ ELISA, we used DK-PGD_2_, a specific DP2 agonist that was undetectable in the assay (Supplemental Fig. 2A), or anti–CD3/CD28 to stimulate the cells (Fig. 6A). Both DK-PGD_2_ and anti–CD3/CD28 strongly induced PGD_2_ production, which peaked after 4-h treatment. The capacity of PGD_2_ production in Tc2 cells (22.3 ± 43.2 ng PGD_2_/1 × 10^6^ cells) was similar to that in Th2 cells (29.3 ± 57.1 ng PGD_2_/1 × 10^6^ cells) (Supplemental Fig. 2B) but was less than half of the capacity of mast cells (51.7 ng PGD_2_/1 × 10^6^ cells) (5). Blockade of DP2 with fevipiprant significantly inhibited the PGD_2_ production triggered by DK-PGD_2_, confirming autocrine or paracrine generation of PGD_2_ in Tc2 cells (Fig. 6B). Interestingly, fevipiprant also significantly reduced the PGD_2_ production induced by TCR.

To confirm the biosynthetic pathway of PGD_2_ in Tc2 cells, we examined the expression of hPGDS, an enzyme required for PGD_2_ synthesis, in Tc2 cells with flow cytometry (Fig. 6C). The frequency of hPGDS-positive Tc2 cells was correlated with blood eosinophil counts in the asthma cohort (Fig. 6D). DK-PGD_2_ but not anti–CD3/CD28 upregulated the expression of hPGDS, which was reversed by fevipiprant (Fig. 6E). The expression of COX-1/2, another group of enzymes required for PGD_2_ synthesis, in Tc2 cells was also examined (Fig. 6F, 6G; Supplemental Fig. 2C, 2D). DK-PGD_2_ slightly upregulated both COX-1 and COX-2 in Tc2 cells at mRNA (*PTGS1* for COX-1 and *PTGS2* for COX-2) and protein levels, which were inhibited by fevipiprant, although the change of COX-1 was difficult to determine with flow cytometry, as all the cells showed COX-1–positive staining. Anti–CD3/CD28 only upregulated COX-2, but this was not affected by fevipiprant. Inhibition of hPGDS with hPGDS inhibitor 1 or COXs with diclofenac or flurbiprofen completely blocked the PGD_2_ production in Tc2 cells induced by DK-PGD_2_ (Fig. 6H). The inhibition of COXs also blocked the PGD_2_ production induced by anti–CD3/CD28. However, the inhibition of hPGDS significantly but only partially inhibited the effect of anti–CD3/CD28 (Fig. 6H). We also examined cytosolic phospholipase A2 (cPLA2) and phospholipase C (PLC), the potential enzymes upstream of COXs required for PGD_2_ synthesis. Both enzymes, but dominantly cPLA2, were expressed in Tc2 cells (Supplemental Fig. 2E) and were not significantly regulated by the DK-PGD_2_ activation of Tc2 cells, although PLA2 was weakly upregulated by TCR activation (Supplemental Fig. 2F). The PLA2 inhibitor ATK strongly inhibited PGD_2_ production, the PLC inhibitor U-73122 only partially reduced PGD_2_ production, and the combination of ATK and U-73122 further enhanced the inhibition (Supplemental Fig. 2G).

**FIGURE 7.**
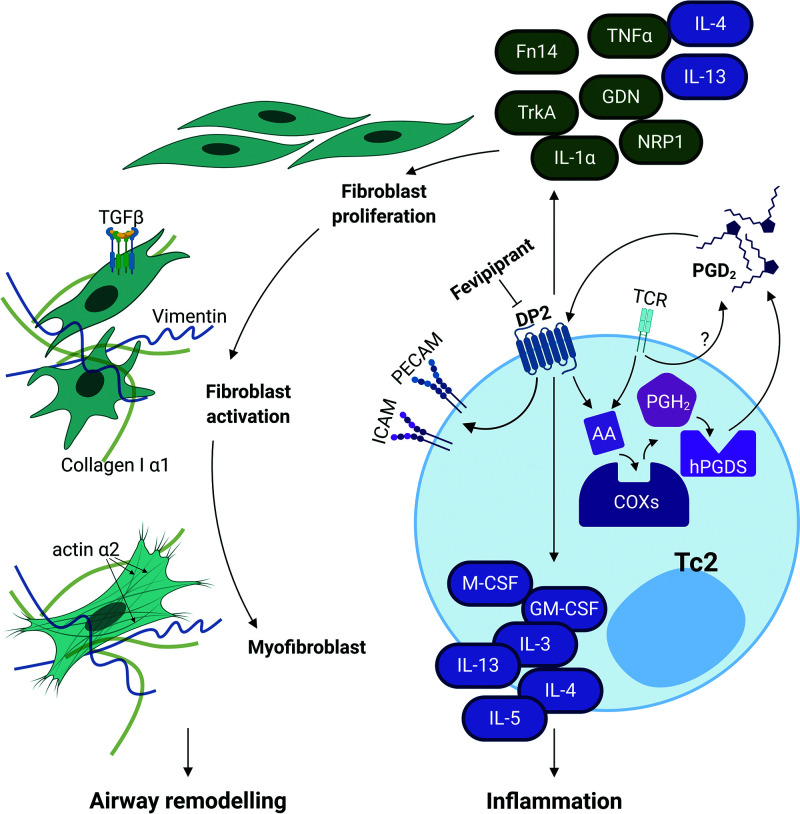
Scheme summarizing the involvement of human Tc2 cells in PGD_2_ autocrine production and tissue remodeling mediated by PGD_2_/DP2 axis. Activation of DP2 by PGD_2_ causes multiple gene upregulations, including proinflammatory cytokines (IL-3/4/5/13, GM-CSF, and M-CSF), adhesion molecules (ICAM and PECAM) and protissue remodeling proteins (IL-1α, Fn14, TrkA, NRP1, GDN, and TNF-α). These protissue remodeling proteins can enhance fibroblast proliferation and activation that also produce protissue remodeling proteins (TGF-β, vimentin, collagen I α1, and actin α2), leading to myofibroblast differentiation. Activation of DP2 also activates PGD_2_ synthesis pathway in Tc2 cells, leading to IgE-independent PGD_2_ autocrine production, which in turn further activate DP2. Fevipiprant, competitive binding to DP2, attenuates both PGD_2_ autocrine and protissue remodeling roles in Tc2 cells. AA, arachidonic acid; GDN, glia-derived nexin; PGH_2_, PG H_2_.

To further confirm the autocrine/paracrine production of PGD_2_ in Tc2 cells, the levels of DP2 endocytosis were examined. Both PGD_2_ and anti–CD3/CD28 induced the loss of DP2 level on the cell surface because of endocytosis (Fig. 6I, 6J). Blockade of DP2 with fevipiprant completely blocked the loss mediated by PGD_2_ and significantly reduced the loss caused by anti–CD3/CD28 (Fig. 6I). Inhibition of COXs and hPGDS reduced the DP2 endocytosis induced by anti–CD3/CD28 but not that by DK-PGD_2_ (Fig. 6J). This was expected, as DP2 on the surface of DK-PGD_2_–treated cells had already been occupied, and inhibition of PGD_2_ synthesis could not reverse this. Furthermore, the inhibition of COXs and hPGDS also mitigated IL-13 production induced by anti–CD3/CD28 (Fig. 6K), indicating the role of PGD_2_ autocrine production in the TCR-mediated Tc2 activation (Fig. 7).

Although the synthesis of cysteinyl leukotrienes (cysLTs) shares the same upstream source of arachidonic acid with PGD_2_, LTE_4_ levels detected in Tc2 cultures were low and not changed by the stimulation of DK-PGD_2_ or anti–CD3/CD28 (Supplemental Fig. 3A). The expression levels of 5-lipoxygenase (5-LO) and 5-LO–activating protein (FLAP), critical proteins required for leukotriene synthesis, in Tc2 cells were also low and not significantly affected by the stimulations (Supplemental Fig. 3B).

## Discussion

Tc2 cells are enriched in both peripheral blood and airways in severe eosinophilic asthma (3). Our previous study has demonstrated that the activation of Tc2 cells could contribute to airway eosinophilia through producing proinflammatory cytokines IL-4/5/13 and GM-CSF. In this study, we revealed some previously, to our knowledge, unrecognized functions of Tc2 cells, including promoting tissue remodeling and IgE-independent PGD_2_ autocrine production, which could play critical roles in the pathogenesis of asthma (Fig. 7). The PGD_2_/DP2 axis enables multi-proinflammatory functions in Tc2 cells, promoting cell recruitment and activation, particularly type 2 cytokine production (3). Using fevipiprant, we demonstrated in this study that competitive inhibition of DP2 not only completely blocked the cell migration, adhesion, proinflammatory cytokine production, and survival functions in Tc2 triggered by PGD_2_ but also attenuated tissue-remodeling effects and PGD_2_ autocrine production in Tc2. These findings will enable a better understanding of the potential role of Tc2 cells in the pathogenesis of asthma.

Airway remodeling, particularly airway wall thickening, is a characteristic feature of asthma (24) that involves structural changes in the airways, including epithelial hyperplasia and metaplasia, subepithelial fibrosis, smooth muscle cell hyperplasia, and angiogenesis, leading to deleterious consequences on lung function. The mechanisms regulating airway remodeling remain poorly understood. It has been suggested that airway remodeling could be regulated by the interaction of immune cells with tissue-forming cells (25). In this study, we demonstrated that PGD_2_ upregulated many proteins produced in Tc2 cells that could play important roles in tissue remodeling. IL-1α, TNF-α, syndecan-4, TrkA, NRP1, and TWEAK/Fn14 are capable of stimulating fibroblast and smooth muscle cell proliferation (26–31). IL-1α, TNF-α and NRP1 promote fibrosis (32–34). NRP1 can play versatile roles in angiogenesis (35). TWEAK/Fn14, glia-derived nexin, and TrkA are able to induce extracellular matrix generation and deposition (36–38). The type 2 cytokines released by activated Tc2 cells can also contribute to tissue remodeling, as it has been demonstrated that tissue injury promotes type 2 cytokine production by the tissue-resident CD8^+^ T cells that promote wound repair in mouse (39). Our data further confirmed this in human cells. The protissue remodeling role of Tc2 cells mediated by the PGD_2_/DP2 axis was also evidenced by our fibroblast–Tc2 coculture assay, in which PGD_2_-activated Tc2 cells enhanced fibroblast proliferation that was mediated by both soluble tissue-remodeling factors in the Tc2 medium and direct cell contact. Activated Tc2 cells also promoted the synthesis of collagen I α1, actin α2, vimentin, and TGF-β in fibroblasts. Collagen I α1 is a member of type 1 collagen, which is the most abundant component of extracellular matrix and can contribute to myofibroblast differentiation (40); actin α2, also named as α smooth muscle actin, is a biomarker of myofibroblasts and smooth muscle differentiation (41); vimentin plays important roles on fibroblast proliferation during wound healing (42), whereas TGF-β signaling controls expression of type 1 collagen and actin α2 (43, 44). In a recent publication, it was reported that fevipiprant reduced ASM mass in asthma patients (45). Although ASM cells were shown to express DP2, DK-PGD_2_ did not directly induce migration of ASM cells in vitro. It was also demonstrated that there was a correlation between ASM mass reduction and the numbers of myofibroblasts or fibrocytes in the lamina propria, which may explain reduced airway remodeling with DP2 antagonism. Our results support these observations and provide a novel, to our knowledge, mechanism for how PGD_2_/DP2 could regulate airway remodeling via activation of Tc2 cells, suggesting a potential strategy to control airway remodeling in asthma (Fig. 7). Of course, further investigation is required to confirm the role of the PGD_2_/DP2/Tc2 axis in human airway remodeling.

PGD_2_ is a major arachidonic acid metabolite detected in high concentrations at sites of allergic inflammation and plays an important role in inflammatory reactions (6, 7). It was traditionally considered that PGD_2_ is predominantly released from activated mast cells during an allergic response initiated by IgE cross-linkage of its high-affinity receptor FcεRI (5). However, high levels of PGD_2_ are not always correlated with the levels of IgE in asthma patients. Increasing evidence suggests that PGD_2_ can also be produced by some FcεRI-low or -negative cells, such as dendritic cells, macrophages (46), eosinophils (47), Th2 cells (8), and ILC2s (48). In this study, we demonstrated for the first time, to our knowledge, that Tc2 cells are capable of producing PGD_2_ via an IgE-independent pathway. The capacity of PGD_2_ production in Tc2 cells is similar to that in Th2 cells and about half of that in mast cells. Tc2 cells possess and use the routine molecular machinery for PGD_2_ synthesis, as inhibition of cPLA2/PLC, COX-1/2, or hPGDS blocked PGD_2_ production in Tc2 cells. PLA2 and PLC are enzymes that cleave membrane phospholipids to arachidonic acid upstream of PGD_2_ synthesis. Our data suggested that, in Tc2 cells, arachidonic acid synthesis was predominantly mediated by cPLA2. COX-1 and COX-2, also known as PG-endoperoxide synthases, convert arachidonic acid to prostanoids consisting of PGs, thromboxanes, and prostacyclins (49). The levels of COX-1/2 in Tc2 cells are upregulated during endogenous synthesis of PGD_2_. hPGDS is a cytosolic enzyme downstream of COXs in the PGD_2_ synthesis pathway that isomerizes PGH_2_, a common precursor for all PGs and thromboxanes, to PGD_2_ in a glutathione-dependent manner (50). Tc2 cells express hPGDS, and hPGDS-positive Tc2 cells are increased in eosinophilic asthma and correlated with blood eosinophil counts in asthma patients. These observations suggest that Tc2 cells could contribute to IgE-independent DP2-mediated airway inflammation in asthma, which could be supported by clinical evidence that ∼20% of severe eosinophilic asthma patients are nonatopic with low IgE levels (51). Stimulation of TCR also strongly promotes PGD_2_ production in Tc2 cells, confirming a TCR-dependent mechanism of PGD_2_ production (8). This response in Tc2 cells is likely involved in secondary autocrine or paracrine production of PGD_2_, as the inhibition of DP2 with fevipiprant markedly attenuated PGD_2_ production induced by TCR stimulation and reversed TCR-mediated DP2 endocytosis, and the inhibition PGD_2_ synthesis also mitigated TCR-mediated cytokine production. In addition to the secondary autocrine synthesis of PGD_2_, TCR activation could also trigger some unknown pathways to regulate PGD_2_ production in Tc2 cells because of the following factors: 1) TCR activation did not upregulate COX-1 or hPGDS, 2) COX-2 upregulation by TCR was not reduced by fevipiprant, and 3) inhibition of DP2 and hPGDS could not completely block the PGD_2_ production induced by TCR activation. Therefore, further investigation is required to understand the complete mechanism involved in TCR-dependent PGD_2_ production.

Both PGD_2_ and cysLTs are major arachidonic acid metabolites released from mast cells, playing critical roles in the pathogenesis of allergic disorders. They are synthesized by separate metabolic pathways downstream of arachidonic acid. In mast cells activated by IgE, PGD_2_ and cysLTs are produced simultaneously, and inhibition of one of their pathways could potentiate the other one (52). However, in activated Tc2 cells, only PGD_2_ but not cysLT production is detected. Considering that very low expression levels of 5-LO and FLAP were detected in Tc2 cells, they might be unable to produce cysLTs because of a lack of enzymes for cysLT synthesis.

DP2 inhibition with fevipiprant has been reported to abolish the proinflammatory effect of PGD_2_ in Th2 and ILC2 cells (20, 21). In this study, we demonstrated that fevipiprant is a potent and specific inhibitor of the DP2 pathway in Tc2 cells. It not only inhibited established Tc2 proinflammatory activation, including migration, adhesion, cytokine production and survival (3), but also suppressed the newly discovered tissue-remodeling effect and autocrine/paracrine PGD_2_ production in Tc2 cells. These findings may be relevant to the design of clinical studies with ongoing DP2 antagonists (13, 14).

Overall, these data expand the array of potential effector functions of type 2 cells, specifically of CD8^+^ T cell populations, that are often overlooked. Additionally, the blockade of these cells and pathways remains of potential clinical value in asthma and other type 2–driven inflammatory diseases.

## Supplementary Material

Data Supplement
