# Supplementary material for: The Roles of Type 2 Cytotoxic T Cells in Inflammation, Tissue Remodeling, and Prostaglandin (PG) D2 Production Are Attenuated by PGD2 Receptor 2 Antagonism
Source: J Immunol. 2021 Jun 1;206(11):2714–24. doi: 10.4049/jimmunol.2001245 (PMC7610864; doi:10.4049/jimmunol.2001245)
Supplement: Data Supplement [file JI_2001245.zip › JI_2001245_Supplemental_1.pdf]

Supplemental Table 1. Primers and probes used for q-PCR

Gene	Primer	Probe No.
ACTA2	5'-CTGTTCCAGCCATCCTTCAT-3'<br>5'-TCATGATGCTGTTGTAGGTGGT-3'	SYBR^{TM} GREEN
AHR	5'-CAACATCACCTACGCCAGTC-3'<br>5'-GCTTGGAAGGATTTGACTTGA-3'	SYBR^{TM} GREEN
ALOX5	5'-CTCAAGCAACACCGACGTAAA-3'<br>5'-CCTTGTGGCATTTGGCATCG-3'	SYBR^{TM} GREEN
ALOX5AP	5'-TTGCCTTTGAGCGGGTCTAC-3'<br>5'-GCCTCACAAACAAGTACATCAGT-3'	SYBR^{TM} GREEN
COL1A1	5'-GGGATTCCCTGGACCTAAAG-3'<br>S'-GGAACACCTCGCTCTCCA-3'	SYBR^{TM} GREEN
CSF1	5'-GCAAGAACTGCAACAACAGC-3'<br>5'-ATCAGGCTTGGTCACCACAT-3'	SYBR^{TM} GREEN
CSF2	5'-TCTCAGAAATGTTTGACCTCCA-3'<br>5'-GCCCTTGAGCTTGGTGAG-3'	SYBR^{TM} GREEN
EDARADD	5'-ACTGCCCACGAAATTCAGATA-3'<br>5'-TGGAAGAGGATCTCCAGTGC-3'	SYBR^{TM} GREEN
EPAS1	5'-GACATGAAGTTCACCTACTGTGATG-3'<br>5'-CGGAGTCTAGCGCATGGTA-3'	SYBR^{TM} GREEN
GAPDH	5'-AGCCACATCGCTCAGACAC-3'<br>5'-GCCCAATACGACCAAATCC-3'	60
HMOX1	5'-GGCAGAGGGTGATAGAAGAGG-3'<br>5'-AGCTCCTGCAACTCCTCAAA-3'	SYBR^{TM} GREEN
HPGDS	5'-GGGGGAGAGAATGGCTTATT-3'<br>5'-CAGGTCAGGCTTAAAGACCAA-3'	SYBR^{TM} GREEN
ICAM	5'-CCTTCCTCACCGTGTACTGG-3'<br>5'-AGCGTAGGGTAAGGTTCTTGC-3'	SYBR^{TM} GREEN
IL1A	5'-CGCCAATGACTCAGAGGAAGA -3'<br>5'-AGGGCGTCATTCAGGATGAA -3'	SYBR^{TM} GREEN
IL3	5'-TTGCCTTTGCTGGACTTCA-3'<br>5'-CTGTTGAATGCCTCCAGGTT-3'	SYBR^{TM} GREEN
IL4	5'-CACCGAGTTGACCGTAACAG-3'<br>5'-GCCCTGCAGAAGGTTTCC-3'	16
IL5	5'-GGTTTGTTGCAGCCAAAGAT-3'<br>5'-TCTTGGCCCTCATTCTCACT-3'	25
IL13	5'-AGCCCTCAGGGAGCTCAT-3'<br>5'-CTCCATACCATGCTGCCATT-3'	17
NRP1	5'-GAGATTATCCTGGAATTTGAAAGC-3'<br>5'-CACAGTAACGCCCAATGTGA-3'	SYBR^{TM} GREEN
NTRK1	5'-CAATGCCTCGGTGGATGT-3'<br>5'-GCAGACCCCCAGATTTCA-3'	SYBR^{TM} GREEN
PECAM	5'-GCAACACAGTCCAGATAGTCGT-3'<br>5'-GACCTCAAACTGGGCATCAT-3'	SYBR^{TM} GREEN
PLA2	5’-CCTCCCCACCCTGAAAAA-3’<br>5’-TGCCAAAATTTTAAATGGAACAC-3’	SYBR^{TM} GREEN
PLAUR	5’-AGCCTTACCGAGGTTGTGTG-3’<br>5’-CTTCGGGAATAGGTGACAGC-3’	SYBR^{TM} GREEN
PLC	5’-CAGTGTTAATGAGAACCACTCCA-3’<br>5’-TTGCTGACTCTCTTCTCTCTTAACC-3’	SYBR^{TM} GREEN
PPARG	5'-GACAGGAAAGACAACAGACAAATC-3'<br>5'-GGGGTGATGTGTTTGAACTTG-3'	SYBR^{TM} GREEN
PRDM1	5'-GTGGTGGGTTAATCGGTTTG-3'<br>5'-GAAGCTCCCCTCTGGAATAGA-3'	SYBR^{TM} GREEN
PTGS1	5'-GGATGGGAAACTCAAGTACCAG-3'<br>5'-AACACAGGCGCCTCTTCTAC-3'	SYBR^{TM} GREEN
PTGS2	5'-CTTCACGCATCAGTTTTTCAAG-3'<br>5'-TCACCGTAAATATGATTTAAGTCCAC-3'	SYBR^{TM} GREEN
SDC4	5'-GGCAGGAATCTGATGACTTTG-3'<br>5'-GGCCGATCATGGAGTCTTC-3'	SYBR^{TM} GREEN
SERPINE2	5'-TCTGCCTGTGATTCCATCAA-3'<br>5'-GGTGAGCACACCATCAATAAGAT-3'	SYBR^{TM} GREEN
TNF	5'-CAGCCTCTTCTCCTTCCTGAT-3'<br>5'-GCCAGAGGGCTGATTAGAGA-3'	SYBR^{TM} GREEN
TNFRSF12A	5'-GACCGCACAGCGACTTCT-3'<br>5'-CACGAAGGTCAGGCTCAGA-3'	SYBR^{TM} GREEN
TNFSF11	5'-TGATTCATGTAGGAGAATTAAACAGG-3'<br>5'-GATGTGCTGTGATCCAACGA-3'	SYBR^{TM} GREEN
VIM	5'-TGGTCTAACGGTTTCCCCTA-3'<br>5'-GACCTCGGAGCGAGAGTG-3'	SYBR^{TM} GREEN

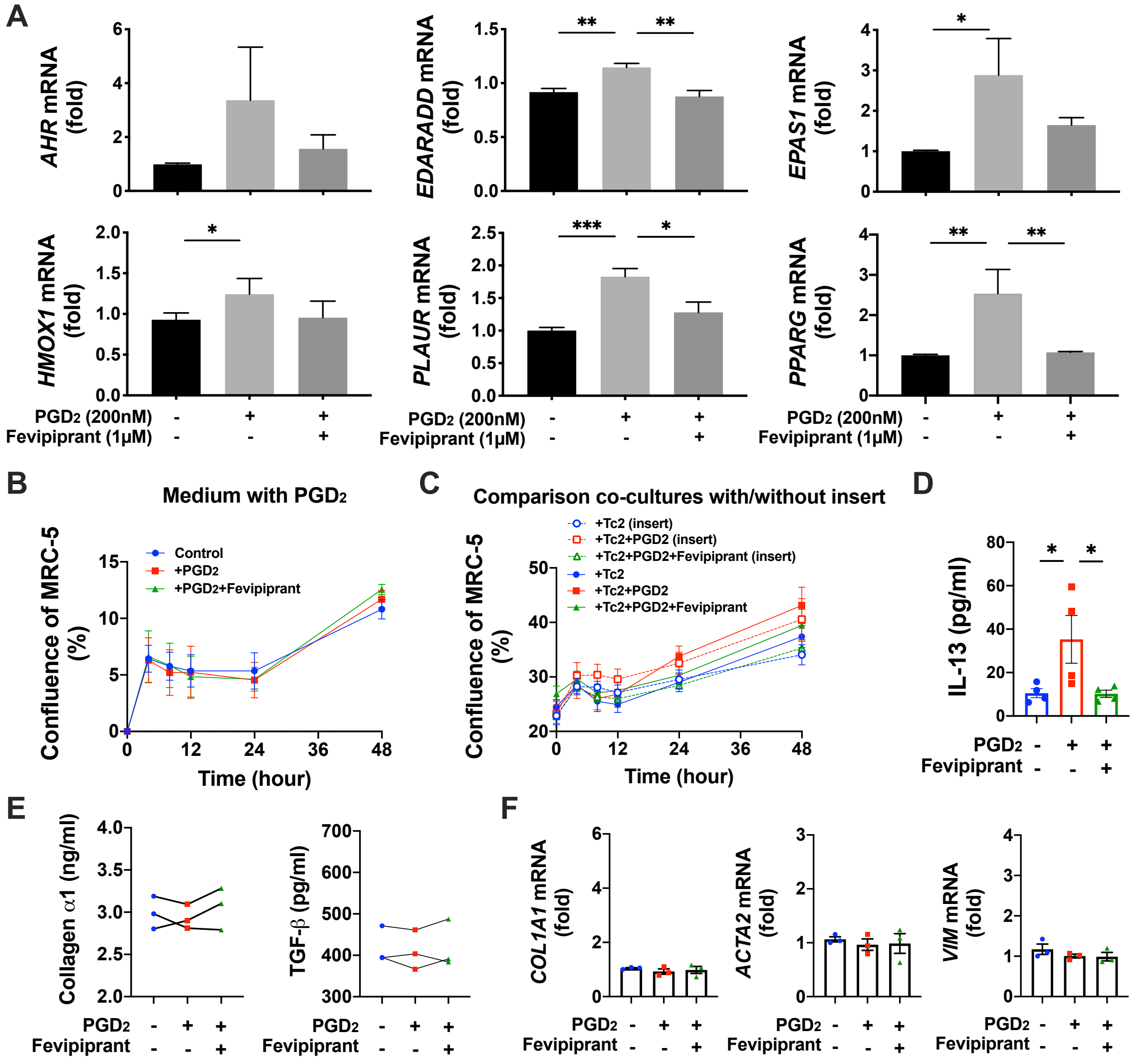

Suppl. Figure 1. Effects of PGD_2 and fevipiprant on Tc2 tissue remodelling genes and MRC5 cell growth. (**A**) The upregulation of tissue remodelling genes by PGD_2 was inhibited by fevipiprant. The mRNA levels were measured with qPCR. (**B**) The confluence of MRC5 cells cultured with medium alone, or medium containing 200 nM PGD_2 in presence or absence of 1 μM fevipiprant. (**C**) Comparison of MRC-5 cell confluence in the MRC-5-Tc2 co-cultures using and without using insert to separate two types of cells. (**D**) Concentration of IL-13 in the supernatants of the co-culture ($5x10^4$ Tc2 cells/ml). (**E**) Protein concentrations of collagen α1 and TGF-β in the supernatants of MRC5 cell cultures under condition **B** detected with ELISA. (**F**) mRNA levels of *COL1A1*, *ACTA2* and *VIM* in MRC-5 cells from **B** measured with qPCR. Data are expressed as mean ± SEM of six to seven (**A**) independent experiments. **p*<0.05; ***p*<0.01, ****p*<0.001.

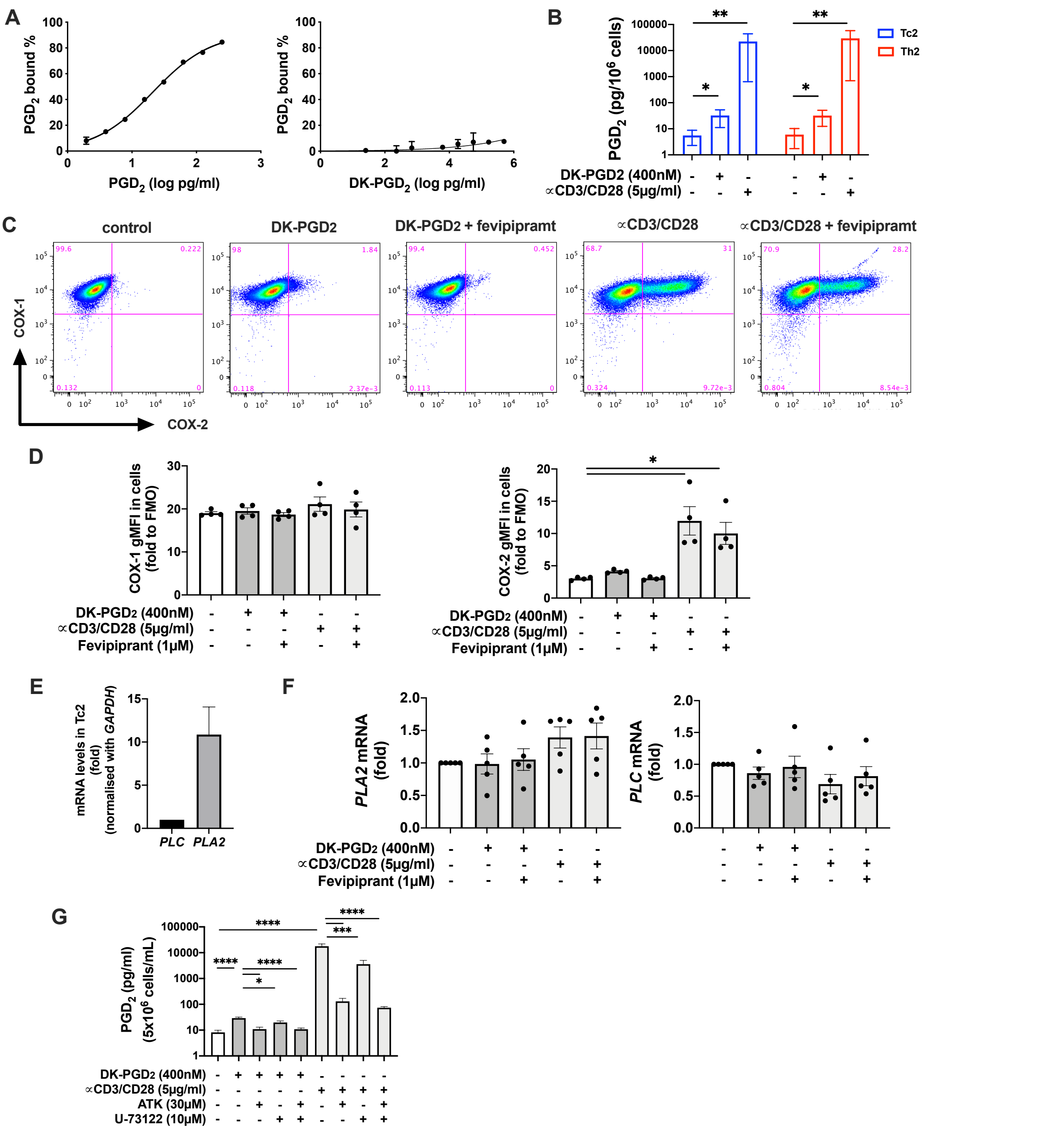

Suppl. Figure 2. PGD_2 production and expression of COX-1/COX-2 and PLA2/PLC after treatments in cultured Tc2 or Th2 cells from Leucocyte Cones. (**A**) PGD_2 or DK-PGD_2 was tested in a PGD_2–MOX enzyme immunoassay kit. (**B**) Comparison of PGD_2 production after treatments with DK-PGD_2 or anti-CD3/CD28 antibodies between cultured Tc2 and Th2 cells from same donors. (**C**) A representative flow cytometric gating example comparing the levels of COX-1 and COX-2 in Tc2 cells after indicated treatments. (**D**) Comparison of the staining levels (gMFI) of COX-1 and COX-2 in Tc2 cells after indicated treatments. (**E**) Comparison of mRNA levels between *PLA2* and *PLC* in Tc2 cells. (**F**) Transcription levels of *PLA2* and *PLC* after indicated treatments. (**G**) PGD_2 production after DK-PGD_2 or anti-CD3/CD28 in the absence or presence ATK or U-73122. Data are expressed as mean ± SEM of one to two (**A**), four (**B**), five (**E**) or seven (**G**) independent experiments. *$p<0.05$, **$p<0.01$, ***$p<0.001$, ****$p<0.0001$.

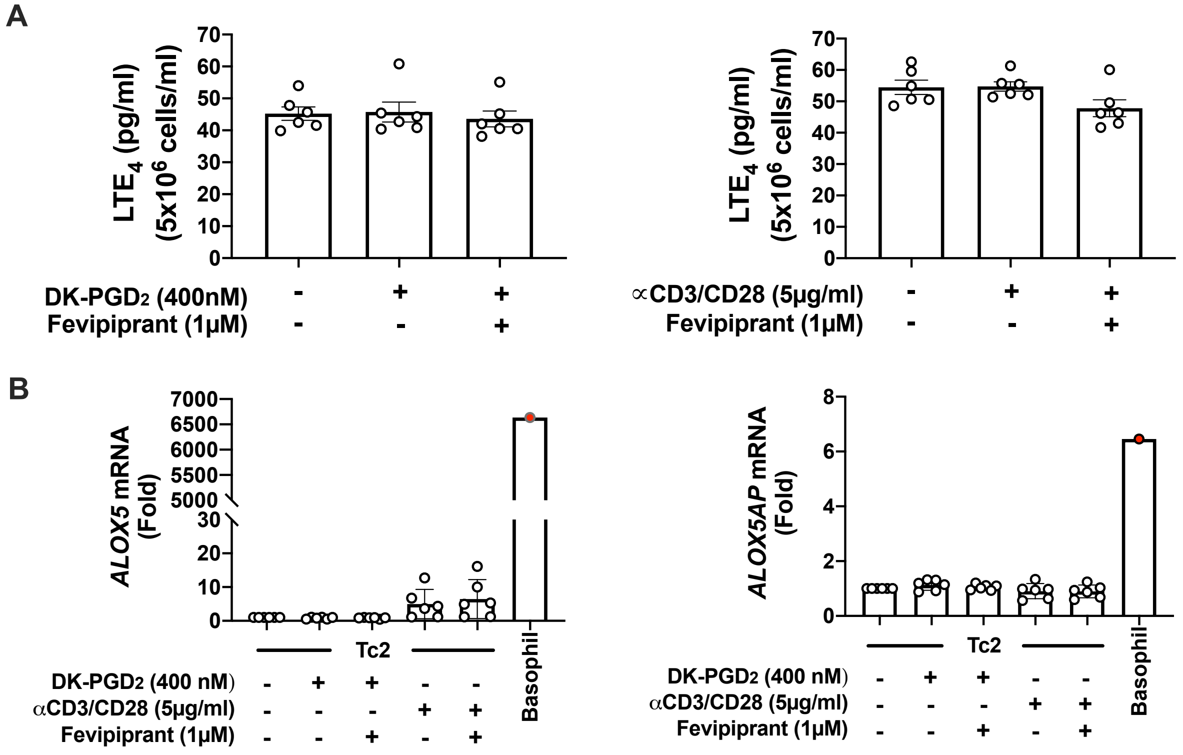

Suppl. Figure 3. Production of LTE_4 and the proteins involved in cysLT synthesis were not significantly affected by Tc2 cell stimulation. Cultured Tc2 cells from Leucocyte Cones were treated with DK-PGD_2 or anti-CD3/CD28 in the presence or absence of fevipiprant for 4 h. (**A**) The concentrations of LTE_4 in the supernatants were measured with MOX enzyme immunoassay. (**B**) The mRNA levels of *ALOX5* (gene for 5-lipoxygenase) and *ALOX5AP* (gene for FLAP) in the cell pellets were determined with qPCR. The RNA from human basophils was used as a positive control. Data are expressed as mean ± SEM.
